# {*N*-[(4-Meth­oxy-2-oxidophen­yl)(phen­yl)methyl­idene]glycinato}di­phenyl­silicon(IV)

**DOI:** 10.1107/S2414314623003061

**Published:** 2023-04-06

**Authors:** Uwe Böhme, Sabine Fels

**Affiliations:** a Institut für Anorganische Chemie, Technische Universität Bergakademie Freiberg, Leipziger Str. 29, 09599 Freiberg, Germany; University of Kentucky, USA

**Keywords:** crystal structure, silicon complex, Schiff-base ligand, penta­coordination

## Abstract

The title compound, C_28_H_23_NO_4_Si, crystallizes in the monoclinic space group *P*2_1_/*c*. The silicon complex consists of a tridentate dinegative Schiff base ligand bound to a di­phenyl­silyl unit. The coordination geometry of the penta­coordinate silicon atom is a distorted trigonal bipyramid.

## Abstract 2

The silicon title complex consists of a tridentate dinegative Schiff base ligand bound to a di­phenyl­silyl unit. The coordination geometry of the penta­coordinated silicon atom is a distorted trigonal bipyramid.

## Graphical abstract

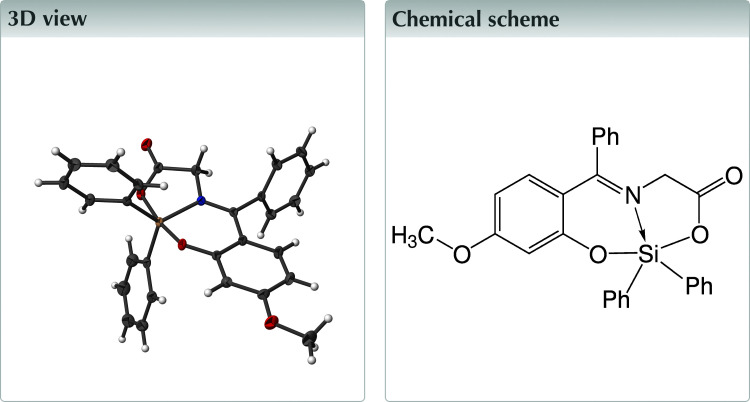

## Structure description

Penta­coordinate silicon complexes can be generated with tridentate *O*,*N*,*O′*-chelate ligands based on Schiff bases (Wagler *et al.*, 2014 ▸). The Schiff base {(*E*)-[(2-hy­droxy-4-meth­oxy­phen­yl)(phen­yl)methyl­idene]amino}­acetic acid has been utilized once previously to prepare a tin complex (Singh *et al.*, 2018 ▸). The tin atom therein is coordinated to the tridentate Schiff-base ligand, two methyl groups and a methanol mol­ecule, resulting in a hexa­coordinate complex. This ligand has not been used so far for the generation of silicon complexes. Related silicon complexes contain Schiff base ligands derived from salicyl aldehyde (Warncke *et al.*, 2012 ▸), aceto­phenone (Böhme *et al.*, 2006 ▸) or naphthyl aldehyde (Schwarzer *et al.*, 2018 ▸).

**Figure 1:**
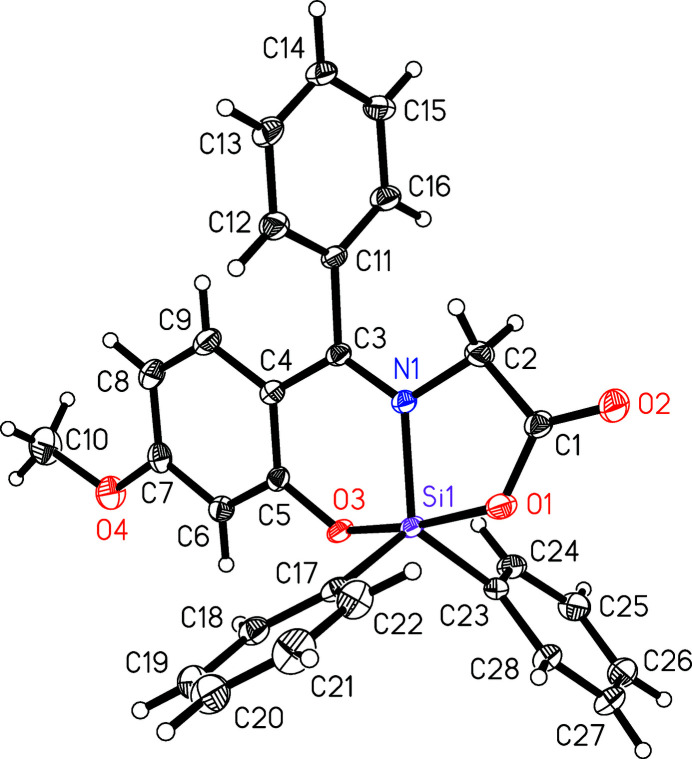
A view of the mol­ecular structure of the title compound, with the atom-labelling scheme. Displacement ellipsoids are drawn at the 50% probability level.

The asymmetric unit of the title compound contains one mol­ecule of {*N*-[(4-meth­oxy-2-oxidophen­yl)(phen­yl)methyl­idene]glycinato}di­phenyl­silicon(IV). The mol­ecular structure is shown in Fig. 1 ▸ (50% displacement ellipsoids). The Schiff base acts as tridentate dinegative ligand. The silicon complex contains a penta­coordinate silicon atom, which is coordinated to the carboxyl-O1, phen­oxy-O3, imine-N1 and two carbon atoms from phenyl groups (C17 and C23). The coordination geometry of the penta­coordinate silicon atom can be analysed with the parameter τ. The parameter is defined as *τ* = (*β* - *α*)/60° with *β* as largest and *α* as the second largest angle at the central atom (Addison *et al.*, 1984 ▸). If τ = 0 it is a perfect square pyramid, while τ = 1 indicates a perfect trigonal bipyramid. The largest angle at the silicon atom is O1—Si1—O3 with 170.83 (4)° and second largest N1—Si1—C23 with 123.23 (5)° (see Table 1 ▸). This leads to a parameter τ = 0.79, which corresponds to a distorted trigonal bipyramid. The apical positions are represented by O1 and O3 of the tridentate ligand, while the atoms N1, C17, and C23 represent the atoms in the trigonal plane.

The bond Si—O1 [1.8361 (10) Å] is longer than the bond Si1—O3 [1.7502 (9) Å]. This can be explained by the carboxyl-type oxygen atom O1 and the electronegative character of the phenyl bound atom O3. The bond lengths for Si1—N1 and Si—C are similar to those in comparable penta­coordinate silicon complexes (Böhme *et al.*, 2006 ▸; Schwarzer *et al.*, 2018 ▸; Böhme & Günther, 2007 ▸; Böhme & Foehn, 2007 ▸). There is one closely related silicon complex with the 2-{(*E*)-[(2-hy­droxy-4-meth­oxy­phen­yl)(phen­yl)methyl­idene]amino}­propanoic acid as ligand (Böhme & Fels, 2023 ▸). The Schiff base ligand therein has an additional methyl group at C2 with an alaninato instead of an glycinato group. The geometric features of that complex are very similar to those of the title compound.

Inter­molecular inter­actions of the title compound are dominated by close-packing. No specific hydrogen bonds can be identified.

## Synthesis and crystallization

The *O*,*N*,*O′*-ligand was prepared from 2-hy­droxy-4-meth­oxy­benzo­phenone and glycine according to a literature procedure (Fels, 2015 ▸). To a solution of 1.1 g (3.9 mmol) of {(*E*)-[(2-hy­droxy-4-meth­oxy­phen­yl)(phen­yl)methyl­idene]amino}­acetic acid in 40 ml of dry THF were added 0.9 g (8.9 mmol) of tri­ethyl­amine and the mixture was cooled to 0°C. 1.0 g (4.0 mmol) of SiCl_2_Ph_2_ was diluted with 20 ml of THF and added *via* a dropping funnel to the solution. The mixture was stirred for 16 h at room temperature. The white precipitate of tri­ethyl­ammonium chloride was separated by filtration. The filtrate was reduced in a vacuum and the pale-yellow residue was dissolved in 20 ml of chloro­form. The resulting suspension was filtered again. 2 ml of *n*-hexane were added to the filtrate and the solution was stored for 6 weeks at 8°C. Pale-yellow crystals suitable for crystal-structure analysis were obtained. Yield: 1.2 g (66%), m.p. = 437 K.

^1^H NMR (400 MHz, CDCl_3_) δ (p.p.m.): 4.10 (*s*, 2H, CH_2_), 4.13 (*s*, 3H, CH_3_—O), 6.37–8.17 (mm, 18H_arom_); ^13^C NMR (101 MHz,CDCl_3_) δ (p.p.m.): 54.5 (CH_2_), 55.9 (CH_3_—O), 102.5, 110.2, 111.9, 125.6, 127.5, 129.3, 129.6, 130.4, 134.0, 135.0, 136.7, 139.9 (12 C_arom_), 167.1, 168.2, 169.1 (C=N, C_arom_—O—Si, C—OMe), 179.1 (COO); ^29^Si NMR (CDCl_3_, 79.5 MHz) δ (p.p.m.): −99.7.

## Refinement

Crystal data, data collection and structure refinement details for the title compound are summarized in Table 2 ▸.

## Supplementary Material

Crystal structure: contains datablock(s) I. DOI: 10.1107/S2414314623003061/pk4039sup1.cif

Structure factors: contains datablock(s) I. DOI: 10.1107/S2414314623003061/pk4039Isup2.hkl

CCDC reference: 2253751

Additional supporting information:  crystallographic information; 3D view; checkCIF report

## Figures and Tables

**Table 1:** Selected geometric parameters (Å, °)

Si1—O3	1.7502 (9)	Si1—C23	1.8817 (13)
Si1—O1	1.8361 (10)	Si1—C17	1.8940 (13)
Si1—N1	1.8726 (11)		
			
O3—Si1—O1	170.83 (5)	N1—Si1—C23	123.23 (5)
O3—Si1—N1	90.36 (4)	O3—Si1—C17	96.56 (5)
O1—Si1—N1	82.80 (4)	O1—Si1—C17	91.93 (5)
O3—Si1—C23	90.38 (5)	N1—Si1—C17	115.75 (5)
O1—Si1—C23	88.28 (5)	C23—Si1—C17	120.51 (5)

**Table 2:** Experimental details

Crystal data	
Chemical formula	C_28_H_23_NO_4_Si
*M*_r_	465.56
Crystal system, space group	Monoclinic, *P*2_1_/*c*
Temperature (K)	150
*a*, *b*, *c* (Å)	10.7366 (3), 9.5341 (4), 22.6716 (7)
β (°)	91.603 (2)
*V* (Å^3^)	2319.84 (14)
*Z*	4
Radiation type	Mo *K*α
μ (mm^−1^)	0.14
Crystal size (mm)	0.34 × 0.34 × 0.17

Data collection	
Diffractometer	Stoe *IPDS* 2
Absorption correction	Integration (*X-RED*; Stoe & Cie, 2009 ▸)
*T*_min_, *T*_max_	0.990, 0.996
No. of measured, independent and observed [*I* > 2σ(*I*)] reflections	40516, 5326, 4713
*R*_int_	0.058
(sin θ/λ)_max_ (Å^−1^)	0.650

Refinement	
*R*[*F* ^2^ > 2σ(*F* ^2^)], *wR*(*F* ^2^), *S*	0.037, 0.095, 1.07
No. of reflections	5326
No. of parameters	308
H-atom treatment	H-atom parameters constrained
Δρ_max_, Δρ_min_ (e Å^−3^)	0.35, −0.31

Computer programs: *X-AREA* (Stoe & Cie, 2009 ▸), *X-RED* (Stoe & Cie, 2009 ▸), *SHELXT* (Sheldrick, 2015*a* ▸), *SHELXL2018* (Sheldrick, 2015*b* ▸), *ORTEP-3 for Windows* (Farrugia, 2012 ▸).

## full crystallographic data

### Crystal data

**Table:**

C_28_H_23_NO_4_Si	*D*_x_ = 1.333 Mg m^−^^3^
*M**_r_* = 465.56	Melting point: 437 K
Monoclinic, *P*2_1_/*c*	Mo *K*α radiation, λ = 0.71073 Å
*a* = 10.7366 (3) Å	Cell parameters from 46011 reflections
*b* = 9.5341 (4) Å	θ = 1.8–29.7°
*c* = 22.6716 (7) Å	µ = 0.14 mm^−^^1^
β = 91.603 (2)°	*T* = 150 K
*V* = 2319.84 (14) Å^3^	Prism, pale yellow
*Z* = 4	0.34 × 0.34 × 0.17 mm
*F*(000) = 976	

### Data collection

**Table:**

Stoe IPDS 2 diffractometer	5326 independent reflections
Radiation source: fine-focus sealed tube	4713 reflections with *I* > 2σ(*I*)
Graphite monochromator	*R*_int_ = 0.058
phi and ω scans	θ_max_ = 27.5°, θ_min_ = 1.8°
Absorption correction: integration (X-RED; Stoe & Cie, 2009)	*h* = −13→13
*T*_min_ = 0.990, *T*_max_ = 0.996	*k* = −12→12
40516 measured reflections	*l* = −29→29

### Refinement

**Table:**

Refinement on *F*^2^	Primary atom site location: structure-invariant direct methods
Least-squares matrix: full	Secondary atom site location: difference Fourier map
*R*[*F*^2^ > 2σ(*F*^2^)] = 0.037	Hydrogen site location: inferred from neighbouring sites
*wR*(*F*^2^) = 0.095	H-atom parameters constrained
*S* = 1.07	*w* = 1/[σ^2^(*F*_o_^2^) + (0.0397*P*)^2^ + 1.3299*P*] where *P* = (*F*_o_^2^ + 2*F*_c_^2^)/3
5326 reflections	(Δ/σ)_max_ < 0.001
308 parameters	Δρ_max_ = 0.35 e Å^−^^3^
0 restraints	Δρ_min_ = −0.30 e Å^−^^3^

### Special details

**Table:**

Geometry. All esds (except the esd in the dihedral angle between two l.s. planes) are estimated using the full covariance matrix. The cell esds are taken into account individually in the estimation of esds in distances, angles and torsion angles; correlations between esds in cell parameters are only used when they are defined by crystal symmetry. An approximate (isotropic) treatment of cell esds is used for estimating esds involving l.s. planes.

### Fractional atomic coordinates and isotropic or equivalent isotropic displacement parameters (Å^2^)

**Table:**

	*x*	*y*	*z*	*U*_iso_*/*U*_eq_	
Si1	0.31392 (3)	0.99714 (4)	0.16974 (2)	0.01342 (9)	
O1	0.36844 (8)	1.11274 (10)	0.22987 (4)	0.0200 (2)	
O2	0.51231 (9)	1.13834 (12)	0.30212 (4)	0.0258 (2)	
O3	0.27872 (8)	0.86736 (10)	0.11728 (4)	0.01574 (18)	
O4	0.27972 (10)	0.61405 (12)	−0.05856 (4)	0.0277 (2)	
N1	0.48371 (9)	0.95425 (11)	0.16594 (5)	0.0139 (2)	
C1	0.47765 (11)	1.09141 (14)	0.25473 (6)	0.0171 (2)	
C2	0.55949 (12)	1.00210 (16)	0.21702 (6)	0.0219 (3)	
H2A	0.631490	1.057425	0.203700	0.026*	
H2B	0.591347	0.920591	0.239905	0.026*	
C3	0.54093 (11)	0.89558 (13)	0.12145 (5)	0.0139 (2)	
C4	0.47505 (11)	0.83159 (13)	0.07284 (5)	0.0156 (2)	
C5	0.34401 (11)	0.81485 (13)	0.07368 (5)	0.0138 (2)	
C6	0.28246 (12)	0.73953 (13)	0.02917 (6)	0.0168 (2)	
H6	0.194714	0.727412	0.030160	0.020*	
C7	0.34862 (13)	0.68184 (14)	−0.01680 (6)	0.0192 (3)	
C8	0.47862 (13)	0.69729 (16)	−0.01858 (6)	0.0234 (3)	
H8	0.523837	0.657862	−0.049956	0.028*	
C9	0.53918 (13)	0.77042 (15)	0.02588 (6)	0.0218 (3)	
H9	0.627165	0.780100	0.024952	0.026*	
C10	0.34222 (17)	0.5619 (2)	−0.10933 (7)	0.0372 (4)	
H10A	0.383379	0.639715	−0.129226	0.056*	
H10B	0.281274	0.518221	−0.136575	0.056*	
H10C	0.404609	0.492187	−0.096810	0.056*	
C11	0.68034 (11)	0.89973 (13)	0.12084 (5)	0.0150 (2)	
C12	0.73492 (12)	1.00221 (14)	0.08643 (6)	0.0202 (3)	
H12	0.684162	1.065073	0.063852	0.024*	
C13	0.86400 (13)	1.01245 (15)	0.08514 (6)	0.0225 (3)	
H13	0.901506	1.083111	0.062021	0.027*	
C14	0.93778 (12)	0.91996 (16)	0.11744 (6)	0.0222 (3)	
H14	1.025965	0.926505	0.116231	0.027*	
C15	0.88319 (12)	0.81768 (16)	0.15161 (6)	0.0229 (3)	
H15	0.934325	0.754512	0.173831	0.027*	
C16	0.75420 (12)	0.80664 (14)	0.15365 (6)	0.0197 (3)	
H16	0.717003	0.736480	0.177145	0.024*	
C17	0.25867 (11)	1.15154 (14)	0.12341 (6)	0.0164 (2)	
C18	0.19408 (12)	1.13196 (14)	0.06931 (6)	0.0195 (3)	
H18	0.179825	1.039201	0.055387	0.023*	
C19	0.15030 (13)	1.24442 (16)	0.03546 (7)	0.0253 (3)	
H19	0.105447	1.227468	−0.000544	0.030*	
C20	0.17187 (15)	1.38089 (16)	0.05406 (7)	0.0302 (3)	
H20	0.142694	1.457667	0.030810	0.036*	
C21	0.23654 (16)	1.40427 (16)	0.10701 (8)	0.0320 (3)	
H21	0.252236	1.497441	0.120079	0.038*	
C22	0.27826 (14)	1.29152 (15)	0.14085 (7)	0.0253 (3)	
H22	0.321670	1.309583	0.177144	0.030*	
C23	0.20681 (11)	0.91139 (14)	0.22325 (5)	0.0153 (2)	
C24	0.20644 (12)	0.76595 (14)	0.23268 (6)	0.0196 (3)	
H24	0.257247	0.707386	0.209467	0.024*	
C25	0.13269 (13)	0.70607 (15)	0.27559 (6)	0.0235 (3)	
H25	0.136403	0.607902	0.282586	0.028*	
C26	0.05394 (13)	0.78922 (16)	0.30807 (6)	0.0246 (3)	
H26	0.003126	0.748295	0.337084	0.030*	
C27	0.04988 (13)	0.93253 (16)	0.29792 (6)	0.0243 (3)	
H27	−0.005887	0.989610	0.319156	0.029*	
C28	0.12701 (12)	0.99305 (14)	0.25682 (6)	0.0192 (3)	
H28	0.125525	1.091853	0.251418	0.023*	

### Atomic displacement parameters (Å^2^)

**Table:**

	*U*^11^	*U*^22^	*U*^33^	*U*^12^	*U*^13^	*U*^23^
Si1	0.00968 (15)	0.01667 (17)	0.01402 (16)	−0.00076 (12)	0.00254 (11)	−0.00194 (12)
O1	0.0130 (4)	0.0261 (5)	0.0210 (5)	0.0006 (4)	0.0010 (3)	−0.0089 (4)
O2	0.0209 (5)	0.0365 (6)	0.0200 (5)	−0.0031 (4)	0.0007 (4)	−0.0107 (4)
O3	0.0122 (4)	0.0194 (4)	0.0159 (4)	−0.0013 (3)	0.0038 (3)	−0.0035 (3)
O4	0.0297 (5)	0.0337 (6)	0.0197 (5)	−0.0019 (4)	−0.0022 (4)	−0.0118 (4)
N1	0.0116 (5)	0.0162 (5)	0.0139 (5)	−0.0009 (4)	0.0008 (4)	−0.0012 (4)
C1	0.0138 (6)	0.0203 (6)	0.0172 (6)	−0.0035 (5)	0.0028 (5)	−0.0024 (5)
C2	0.0146 (6)	0.0325 (7)	0.0185 (6)	0.0025 (5)	−0.0025 (5)	−0.0089 (5)
C3	0.0121 (5)	0.0142 (5)	0.0156 (6)	0.0005 (4)	0.0033 (4)	0.0029 (4)
C4	0.0142 (6)	0.0176 (6)	0.0151 (6)	0.0003 (4)	0.0025 (4)	−0.0008 (5)
C5	0.0151 (5)	0.0134 (5)	0.0129 (5)	0.0005 (4)	0.0026 (4)	0.0018 (4)
C6	0.0168 (6)	0.0169 (6)	0.0168 (6)	−0.0014 (5)	0.0007 (5)	−0.0004 (5)
C7	0.0251 (7)	0.0179 (6)	0.0145 (6)	0.0000 (5)	−0.0013 (5)	−0.0013 (5)
C8	0.0240 (7)	0.0295 (7)	0.0170 (6)	0.0030 (6)	0.0053 (5)	−0.0053 (5)
C9	0.0176 (6)	0.0292 (7)	0.0189 (6)	0.0007 (5)	0.0054 (5)	−0.0035 (5)
C10	0.0427 (9)	0.0461 (10)	0.0225 (7)	0.0060 (8)	−0.0027 (7)	−0.0186 (7)
C11	0.0113 (5)	0.0181 (6)	0.0156 (6)	0.0001 (4)	0.0031 (4)	−0.0021 (5)
C12	0.0172 (6)	0.0221 (6)	0.0215 (6)	0.0001 (5)	0.0023 (5)	0.0043 (5)
C13	0.0186 (6)	0.0267 (7)	0.0225 (7)	−0.0071 (5)	0.0054 (5)	0.0032 (5)
C14	0.0123 (6)	0.0329 (7)	0.0215 (6)	−0.0033 (5)	0.0027 (5)	−0.0024 (6)
C15	0.0145 (6)	0.0290 (7)	0.0250 (7)	0.0031 (5)	0.0002 (5)	0.0045 (6)
C16	0.0151 (6)	0.0208 (6)	0.0234 (6)	0.0002 (5)	0.0036 (5)	0.0045 (5)
C17	0.0121 (5)	0.0188 (6)	0.0186 (6)	−0.0006 (4)	0.0064 (4)	0.0006 (5)
C18	0.0165 (6)	0.0210 (6)	0.0210 (6)	−0.0008 (5)	0.0045 (5)	0.0013 (5)
C19	0.0241 (7)	0.0281 (7)	0.0238 (7)	0.0019 (6)	0.0028 (5)	0.0057 (6)
C20	0.0335 (8)	0.0233 (7)	0.0342 (8)	0.0048 (6)	0.0074 (6)	0.0098 (6)
C21	0.0410 (9)	0.0186 (7)	0.0369 (9)	−0.0004 (6)	0.0081 (7)	0.0008 (6)
C22	0.0290 (7)	0.0213 (7)	0.0258 (7)	−0.0027 (5)	0.0033 (6)	−0.0022 (6)
C23	0.0104 (5)	0.0211 (6)	0.0142 (5)	0.0007 (4)	0.0000 (4)	0.0000 (5)
C24	0.0147 (6)	0.0216 (6)	0.0226 (6)	0.0022 (5)	0.0023 (5)	0.0011 (5)
C25	0.0197 (6)	0.0232 (7)	0.0276 (7)	−0.0009 (5)	0.0017 (5)	0.0072 (6)
C26	0.0188 (6)	0.0331 (8)	0.0222 (7)	−0.0033 (5)	0.0055 (5)	0.0069 (6)
C27	0.0190 (6)	0.0318 (8)	0.0225 (7)	0.0015 (5)	0.0084 (5)	−0.0007 (6)
C28	0.0168 (6)	0.0215 (6)	0.0195 (6)	0.0004 (5)	0.0046 (5)	−0.0004 (5)

### Geometric parameters (Å, º)

**Table:**

Si1—O3	1.7502 (9)	C12—C13	1.3905 (18)
Si1—O1	1.8361 (10)	C12—H12	0.9500
Si1—N1	1.8726 (11)	C13—C14	1.382 (2)
Si1—C23	1.8817 (13)	C13—H13	0.9500
Si1—C17	1.8940 (13)	C14—C15	1.386 (2)
O1—C1	1.3026 (15)	C14—H14	0.9500
O2—C1	1.2128 (16)	C15—C16	1.3910 (18)
O3—C5	1.3257 (15)	C15—H15	0.9500
O4—C7	1.3499 (16)	C16—H16	0.9500
O4—C10	1.4371 (18)	C17—C18	1.4044 (18)
N1—C3	1.3201 (16)	C17—C22	1.4062 (19)
N1—C2	1.4693 (16)	C18—C19	1.3926 (19)
C1—C2	1.5066 (18)	C18—H18	0.9500
C2—H2A	0.9900	C19—C20	1.385 (2)
C2—H2B	0.9900	C19—H19	0.9500
C3—C4	1.4295 (17)	C20—C21	1.388 (2)
C3—C11	1.4977 (16)	C20—H20	0.9500
C4—C9	1.4102 (18)	C21—C22	1.388 (2)
C4—C5	1.4166 (17)	C21—H21	0.9500
C5—C6	1.3905 (17)	C22—H22	0.9500
C6—C7	1.3910 (18)	C23—C28	1.3986 (17)
C6—H6	0.9500	C23—C24	1.4030 (18)
C7—C8	1.4052 (19)	C24—C25	1.3936 (19)
C8—C9	1.3741 (19)	C24—H24	0.9500
C8—H8	0.9500	C25—C26	1.386 (2)
C9—H9	0.9500	C25—H25	0.9500
C10—H10A	0.9800	C26—C27	1.386 (2)
C10—H10B	0.9800	C26—H26	0.9500
C10—H10C	0.9800	C27—C28	1.3893 (19)
C11—C12	1.3901 (18)	C27—H27	0.9500
C11—C16	1.3917 (18)	C28—H28	0.9500
			
O3—Si1—O1	170.83 (5)	C12—C11—C3	117.43 (11)
O3—Si1—N1	90.36 (4)	C16—C11—C3	122.20 (11)
O1—Si1—N1	82.80 (4)	C11—C12—C13	119.81 (12)
O3—Si1—C23	90.38 (5)	C11—C12—H12	120.1
O1—Si1—C23	88.28 (5)	C13—C12—H12	120.1
N1—Si1—C23	123.23 (5)	C14—C13—C12	120.07 (13)
O3—Si1—C17	96.56 (5)	C14—C13—H13	120.0
O1—Si1—C17	91.93 (5)	C12—C13—H13	120.0
N1—Si1—C17	115.75 (5)	C13—C14—C15	120.02 (12)
C23—Si1—C17	120.51 (5)	C13—C14—H14	120.0
C1—O1—Si1	119.47 (8)	C15—C14—H14	120.0
C5—O3—Si1	131.68 (8)	C14—C15—C16	120.60 (13)
C7—O4—C10	117.93 (12)	C14—C15—H15	119.7
C3—N1—C2	118.34 (10)	C16—C15—H15	119.7
C3—N1—Si1	127.02 (9)	C15—C16—C11	119.13 (12)
C2—N1—Si1	114.47 (8)	C15—C16—H16	120.4
O2—C1—O1	125.16 (12)	C11—C16—H16	120.4
O2—C1—C2	122.80 (12)	C18—C17—C22	115.98 (12)
O1—C1—C2	112.02 (11)	C18—C17—Si1	121.36 (10)
N1—C2—C1	107.69 (10)	C22—C17—Si1	122.66 (10)
N1—C2—H2A	110.2	C19—C18—C17	121.99 (13)
C1—C2—H2A	110.2	C19—C18—H18	119.0
N1—C2—H2B	110.2	C17—C18—H18	119.0
C1—C2—H2B	110.2	C20—C19—C18	120.30 (14)
H2A—C2—H2B	108.5	C20—C19—H19	119.8
N1—C3—C4	122.62 (11)	C18—C19—H19	119.8
N1—C3—C11	118.84 (11)	C19—C20—C21	119.30 (14)
C4—C3—C11	118.51 (11)	C19—C20—H20	120.3
C9—C4—C5	118.01 (12)	C21—C20—H20	120.3
C9—C4—C3	121.13 (11)	C22—C21—C20	119.98 (14)
C5—C4—C3	120.52 (11)	C22—C21—H21	120.0
O3—C5—C6	119.05 (11)	C20—C21—H21	120.0
O3—C5—C4	120.90 (11)	C21—C22—C17	122.43 (14)
C6—C5—C4	120.03 (11)	C21—C22—H22	118.8
C5—C6—C7	120.38 (12)	C17—C22—H22	118.8
C5—C6—H6	119.8	C28—C23—C24	117.57 (12)
C7—C6—H6	119.8	C28—C23—Si1	120.25 (10)
O4—C7—C6	115.69 (12)	C24—C23—Si1	122.15 (10)
O4—C7—C8	123.73 (12)	C25—C24—C23	121.02 (13)
C6—C7—C8	120.56 (12)	C25—C24—H24	119.5
C9—C8—C7	118.83 (12)	C23—C24—H24	119.5
C9—C8—H8	120.6	C26—C25—C24	120.24 (13)
C7—C8—H8	120.6	C26—C25—H25	119.9
C8—C9—C4	122.18 (12)	C24—C25—H25	119.9
C8—C9—H9	118.9	C25—C26—C27	119.50 (13)
C4—C9—H9	118.9	C25—C26—H26	120.3
O4—C10—H10A	109.5	C27—C26—H26	120.3
O4—C10—H10B	109.5	C26—C27—C28	120.28 (13)
H10A—C10—H10B	109.5	C26—C27—H27	119.9
O4—C10—H10C	109.5	C28—C27—H27	119.9
H10A—C10—H10C	109.5	C27—C28—C23	121.30 (13)
H10B—C10—H10C	109.5	C27—C28—H28	119.3
C12—C11—C16	120.36 (11)	C23—C28—H28	119.3
			
N1—Si1—O1—C1	−18.70 (10)	N1—C3—C11—C12	−98.98 (14)
C23—Si1—O1—C1	105.11 (10)	C4—C3—C11—C12	79.26 (15)
C17—Si1—O1—C1	−134.41 (10)	N1—C3—C11—C16	80.30 (16)
N1—Si1—O3—C5	−28.46 (11)	C4—C3—C11—C16	−101.47 (15)
C23—Si1—O3—C5	−151.69 (11)	C16—C11—C12—C13	−0.5 (2)
C17—Si1—O3—C5	87.53 (11)	C3—C11—C12—C13	178.79 (12)
O3—Si1—N1—C3	24.52 (11)	C11—C12—C13—C14	0.7 (2)
O1—Si1—N1—C3	−161.61 (11)	C12—C13—C14—C15	−0.6 (2)
C23—Si1—N1—C3	115.21 (11)	C13—C14—C15—C16	0.2 (2)
C17—Si1—N1—C3	−72.95 (12)	C14—C15—C16—C11	0.1 (2)
O3—Si1—N1—C2	−160.30 (9)	C12—C11—C16—C15	0.1 (2)
O1—Si1—N1—C2	13.57 (9)	C3—C11—C16—C15	−179.16 (12)
C23—Si1—N1—C2	−69.60 (11)	O3—Si1—C17—C18	8.59 (11)
C17—Si1—N1—C2	102.24 (10)	O1—Si1—C17—C18	−174.89 (10)
Si1—O1—C1—O2	−162.69 (11)	N1—Si1—C17—C18	102.18 (11)
Si1—O1—C1—C2	18.35 (15)	C23—Si1—C17—C18	−85.74 (11)
C3—N1—C2—C1	167.96 (11)	O3—Si1—C17—C22	−171.99 (11)
Si1—N1—C2—C1	−7.67 (14)	O1—Si1—C17—C22	4.53 (11)
O2—C1—C2—N1	175.01 (13)	N1—Si1—C17—C22	−78.40 (12)
O1—C1—C2—N1	−5.99 (16)	C23—Si1—C17—C22	93.67 (12)
C2—N1—C3—C4	172.82 (12)	C22—C17—C18—C19	−1.02 (19)
Si1—N1—C3—C4	−12.16 (18)	Si1—C17—C18—C19	178.44 (10)
C2—N1—C3—C11	−9.02 (17)	C17—C18—C19—C20	1.3 (2)
Si1—N1—C3—C11	166.00 (9)	C18—C19—C20—C21	−0.6 (2)
N1—C3—C4—C9	179.71 (12)	C19—C20—C21—C22	−0.3 (2)
C11—C3—C4—C9	1.54 (18)	C20—C21—C22—C17	0.6 (2)
N1—C3—C4—C5	−7.05 (19)	C18—C17—C22—C21	0.1 (2)
C11—C3—C4—C5	174.79 (11)	Si1—C17—C22—C21	−179.37 (12)
Si1—O3—C5—C6	−163.06 (9)	O3—Si1—C23—C28	−137.42 (10)
Si1—O3—C5—C4	18.54 (17)	O1—Si1—C23—C28	51.65 (10)
C9—C4—C5—O3	178.59 (12)	N1—Si1—C23—C28	131.89 (10)
C3—C4—C5—O3	5.14 (18)	C17—Si1—C23—C28	−39.57 (12)
C9—C4—C5—C6	0.20 (18)	O3—Si1—C23—C24	44.86 (11)
C3—C4—C5—C6	−173.25 (12)	O1—Si1—C23—C24	−126.07 (11)
O3—C5—C6—C7	−179.28 (11)	N1—Si1—C23—C24	−45.83 (13)
C4—C5—C6—C7	−0.87 (19)	C17—Si1—C23—C24	142.71 (10)
C10—O4—C7—C6	175.29 (13)	C28—C23—C24—C25	−2.43 (19)
C10—O4—C7—C8	−3.7 (2)	Si1—C23—C24—C25	175.35 (10)
C5—C6—C7—O4	−178.26 (12)	C23—C24—C25—C26	2.8 (2)
C5—C6—C7—C8	0.8 (2)	C24—C25—C26—C27	−0.5 (2)
O4—C7—C8—C9	178.94 (13)	C25—C26—C27—C28	−2.0 (2)
C6—C7—C8—C9	0.0 (2)	C26—C27—C28—C23	2.4 (2)
C7—C8—C9—C4	−0.7 (2)	C24—C23—C28—C27	−0.17 (19)
C5—C4—C9—C8	0.6 (2)	Si1—C23—C28—C27	−177.99 (11)
C3—C4—C9—C8	173.98 (13)		
